# Genetic Polymorphism of *IL-1A*, *IL-1B* and *TNFA* Predicting the Presence of Periodontopathogenic Bacteria

**DOI:** 10.3390/jcm15041646

**Published:** 2026-02-22

**Authors:** Nina Kalajzic, Ajka Pribisalic, Marina Adriana Jezina Buselic, Samra Prentic Bakic, Dunja Petricic, Ferdinand Josip Buselic, Davorka Sutlovic, Sendi Kuret

**Affiliations:** 1 Faculty of Health Sciences, University of Split, 21000 Split, Croatia; nkalajzic@fzz.unist.hr (N.K.); dsutlovic@fzz.unist.hr (D.S.); 2 Dental Care Croatia, 21000 Split, Croatiadunjapetricic@gmail.com (D.P.); 3 Department of Dental Medicine, School of Medicine, University of Split, 21000 Split, Croatia; 4 Department of Toxicology and Pharmacogenetics, School of Medicine, University of Split, 21000 Split, Croatia

**Keywords:** periodontal disease, periodontopathogenic bacteria, *IL-1A*, *IL-1B*, *TNFA* genetic polymorphism

## Abstract

**Background/Objectives:** Periodontitis is a chronic inflammatory disease characterized by complex interactions between periodontal pathogens and the host immune response. Pro-inflammatory cytokines, particularly interleukins, may influence bacterial colonization and disease expression, but their association with specific periodontal pathogens remains unclear. This study investigated the associations between single-nucleotide polymorphisms in *IL-1A*, *IL-1B*, and *TNFA* and the presence of key periodontopathogenic bacteria in patients from Croatia. **Methods**: A cross-sectional study included 63 participants. Genotypes were determined, and subgingival plaque samples were analyzed for *Aggregatibacter actinomycetemcomitans*, *Porphyromonas gingivalis*, *Prevotella intermedia*, *Tannerella forsythia*, and *Treponema denticola* using real-time PCR. Multivariable logistic regression models assessed associations between cytokine gene polymorphisms and periodontopathogenic bacteria presence, adjusting for age, gender, smoking status, and the presence of systemic diseases. **Results**: Among participants (median age 57.0 years, IQR 43.5–67.0; 58.7% female), *P. intermedia* (87.3%), *T. forsythia* (85.7%), and *T. denticola* (69.8%) were the most prevalent pathogens. The *IL-1A* CC genotype significantly increased the odds of *P. gingivalis* (OR = 5.54; *p* = 0.009) and *T. denticola* (OR = 3.77; *p* = 0.041) presence. The *IL-1B* CC genotype was independently associated with *T. forsythia* (OR = 8.48; *p* = 0.026). No significant associations were observed for *TNFA* polymorphism. Model performance ranged from moderate to good (AUC up to 0.89). **Conclusions**: Genetic variants in *IL-1A* and *IL-1B* may influence periodontal bacterial colonization, while demographic and lifestyle factors showed limited impact. Further studies in larger cohorts are warranted.

## 1. Introduction

Periodontal disease is a chronic inflammatory condition affecting the tissues supporting the teeth and is characterized by progressive destruction of the periodontal ligament and alveolar bone. If left untreated, this process may ultimately result in tooth loss and impaired oral function [1,2]. Periodontal disease is multifactorial in nature and develops as a consequence of dysregulated host immune response to periodontopathogenic bacteria colonizing the oral cavity [3].

Although periodontal disease is considered an infection-related condition, its onset and progression depend on complex interactions between microbial, environmental and host-related factors, with specific periodontopathogenic bacteria identified as crucial etiological agents [4]. The presence of specific periodontopathogenic bacteria is essential for disease initiation. The most prevalent species detected in subgingival plaque include *Aggregatibacter actinomycetemcomitans* (*A. actinomycetemcomitans*), *Porphyromonas gingivalis* (*P. gingivalis*), *Treponema denticola* (*T. denticola*), *Tannerella forsythia* (*T. forsythia*), *Treponema denticola* (*T. denticola*), and *Prevotella intermedia* (*P. intermedia*). These predominately Gram-negative anaerobic bacteria (with *A. actinomycetemcomitans* being facultatively anaerobic) express numerous virulence factors, such as lipopolysaccharides, proteolytic enzymes, fimbriae, leukotoxins, and immunomodulating proteins, which facilitate bacterial adhesion, invasion, and immunomodulation [3,5,6,7].

The bacterium *A. actinomycetemcomitans* is often considered separately, and its presence is associated with three- to four-fold faster bone resorption compared to other periodontopathogens [1,7]. Its presence is often associated with aggressive forms of periodontal disease, but it can also be detected in chronic conditions [8]. This facultative anaerobe possesses a variety of virulence mechanisms, of which the most notable is the production of leukotoxin, which particularly targets the host’s immune cells, contributing to immune system dysregulation and periodontal tissue damage [3,8].

The red complex bacteria—*P. gingivalis*, *T. denticola*, and *T. forsythia*—are strongly associated with advanced periodontal disease and severe periodontal tissue destruction. These bacteria are associated with the formation of subgingival plaque and deep periodontal pockets, as well as bleeding on probing and clinical attachment loss. Furthermore, research indicates that these periodontopathogens exhibit a synergistic effect and cause the host immune response, both innate and adaptive immunity, resulting in the secretion of cytokines, such as interleukin-1 alpha (IL-1α), interleukin-1 beta (IL-1β) and tumor necrosis factor-alpha (TNF-α) [1,2,3,9].

Within this group, *P. gingivalis* is regarded as a key pathogen due to its capacity to alter the host immune response and promote dysbiosis in the oral cavity, causing tissue damage and chronic inflammation [10,11]. *T. denticola*, a highly mobile spirochete, contributes to periodontal destruction through proteolytic activity, disruption of epithelial cell membranes and direct interaction with immune cells, whereas *T. forsythia* produces proteases that evade the host’s immune response [9,12].

The *Prevotella* genus has high diversity, and some of the 30 subspecies, including *P. intermedia*, are members of the orange complex. *P. intermedia* produces a capsule that impairs phagocytosis and enhances intercellular survival, thereby contributing to persistent infection [9,13].

Although the presence of periodontopathogens is one of the necessary factors for disease development, it is evident that the presence of microorganisms alone cannot entirely explain the heterogeneity in disease susceptibility, severity, and progression observed among individuals. Therefore, periodontal disease is currently considered to be caused by interactions between periodontopathogenic bacteria, environmental factors, the host immune system, and particularly genetic variants that can modulate inflammatory and immune system responses [2,14].

Genetic variants, i.e., single-nucleotide polymorphisms (SNPs) in several inflammatory cytokine genes, including *IL-1A*, *IL-1B*, and *TNFA*, have been studied and are associated with the progression of periodontal disease [15]. Moreover, genome-wide association studies suggest that host genetic variability may also influence the composition of the subgingival microbiota [16,17].

One of the most studied pro-inflammatory cytokines associated with periodontal disease is IL-1. The human *IL-1* genes are located on the proximal part of the long arm of chromosome 2 and encode IL-1α and IL-1β inflammatory cytokines [15]. A meta-analysis by Feng et al., including 1356 patients with chronic periodontitis and 249 controls, demonstrated an association between the *IL-1A* (−889 C/T) polymorphism and susceptibility to chronic periodontitis in European populations [18]. Additionally, a Spanish study reported a significant association between *IL-1A* polymorphism and increased *P. intermedia* levels [19]. Pani et al. observed a statistically significant association between IL-1β (+3954 C/T) polymorphism and red complex bacteria, accompanied by elevated IL-1β levels in gingival crevicular fluid [20]. However, other studies failed to confirm these associations in European populations, highlighting inconsistent findings [21,22].

TNF-α is another key pro-inflammatory cytokine, primarily mediated by monocytes and macrophages during the early host immune response to bacterial colonization [23]. The *TNFA* gene has an important role in amplifying inflammatory cascades, inducing other cytokines such as IL-1, and regulating cell proliferation, necrosis, and apoptosis [24]. The *TNFA* (−308 G/A) polymorphism has been associated with the pathogenesis of aggressive periodontitis, contributing to the over-release of other enzymes, such as proteases, leading to alveolar bone resorption [24,25].

Overall, previous research reported inconsistent findings regarding associations between genetic variants in *IL-1A*, *IL-1B* and *TNFA* genes and either the presence of periodontopathogenic bacteria or susceptibility to periodontitis in different populations. Notably, to our knowledge, only a limited number of studies have directly examined the relationship between periodontopathogenic bacteria and cytokine gene polymorphism, and none have been conducted in the Croatian population. Therefore, our study aimed to investigate the association between the presence of five key periodontopathogenic bacteria and SNPs in *IL-1A* (−889 C/T), *IL-1B* (+3954 C/T; in the literature, it is also referred to +3953 C/T) [26] and *TNFA* (−308 G/A) genes, to evaluate their potential role as genetic risk markers for periodontal disease.

## 2. Materials and Methods

### 2.1. Patients and Sample Collection

A total of 63 patients diagnosed with chronic periodontitis (according to the American Academy of Periodontology and European Federation of Periodontology) [27] and positive for at least one periodontopathogenic bacterium were included in this study. None of the patients have received any antimicrobial medications at least three months before the enrollment in the study. Dental examination and microbiological sampling were performed by the same dentist in a private dental office in Split, Croatia. A standardized periodontal examination was performed for all participants to objectively assess oral inflammatory status and periodontal tissue condition. Clinical parameters included gingival index (Löe and Silness), plaque index (Silness and Löe), probing depth (PD), bleeding on probing (BOP), and clinical attachment level (CAL). The diagnosis of periodontitis was made for patients who exhibited PD of ≥4 mm and CAL of 3–4 mm.

Sample collection and analysis were obtained between June 2024 and October 2025. Data for each individual were collected from medical documentation. Pooling method was used for harvesting bacteria from the pocket using 6 paper points of moderate strength (average size, # 30–50) and “pooling” several probes from various pockets equally distributed in both jaws, following which the paper point (samples) would be placed in a cold transport container, delivered to the laboratory and kept at a temperature of +4 °C until the start of the analysis.

All participants provided written informed consent prior to inclusion in the study, agreeing to the use of their personal and clinical data for research purposes. Information regarding systemic conditions, including cardiovascular diseases, diabetes mellitus, and rheumatoid arthritis, as well as smoking status and snoring, was obtained through a structured questionnaire completed at the time of consent. To minimize potential confounding, patients were additionally questioned about current medication use, dietary habits and lifestyle factors.

### 2.2. Detection of Periodontopathogenic Bacteria

#### 2.2.1. Microbiological Sampling

Cotton rolls were used to segregate the sampling area. The tooth surface was cleaned with 70% ethanol and dried with sterile cotton swabs. Samples were obtained from the diseased areas’ deepest pockets with five sterile paper points inserted into the gingival crevice for 15 s and then placed in a sterile 1.5 mL vial for molecular analysis. The samples were sent to a molecular laboratory and had 7-day stability at temperatures ranging from 2 to 8 °C.

#### 2.2.2. DNA Extraction from Periodontal Sample

To extract the DNA from the periodontal sample, 500 µL of saline was added to the tube and mixed vigorously for 10 s to remove bacterial cells from the paper points. The DNA was then extracted using the NucleoSpin^®^-Microbial kit (Macherey-Nagel, Duren, Germany) according to the manufacturer’s instructions. The quantity of extracted DNA was quantified spectrophotometrically at 260 nm with a NanoDrop ND-1000 Spectrophotometer (Thermo Fisher Scientific, Waltham, MA, USA), and its purity was calculated as the ratio of absorbance observed at 260 and 280 nm.

#### 2.2.3. Real-Time PCR for Bacteria Detection

Real-time PCR was carried out using a real-time analyzer, the ABI Prism 7500 Real-Time PCR System (Applied Biosystems, Waltham, MA, USA). The primers for *A. actinomycetemcomitans*, *P. gingivalis*, *P. intermedia*, *T. denticola*, and *T. forsythia* were designed as previously described [28].

The real-time PCR was performed in a total reaction volume of 50 µL, which included 5.0 µL of isolated DNA as the template, 25 µL of Power SYBR Green PCR Master Mix (Thermo Fisher Scientific, Waltham, MA, USA), 18 µL of sterile water, and 2 µL (20 µM) of a bacteria-specific primer pair. The primer concentrations were the same for all assays. Negative and positive controls were included in each batch of specimens. Negative controls contained ultrapure water instead of sample DNA. The positive control consisted of the genomic DNA of the five positive targeted bacteria. The 5.0 µL of negative and 5.0 µL of positive control were included in each analysis run. All amplifications and detections were carried out in a MicroAmp optical 96-well reaction plate. The cycling conditions were initial denaturation at 95 °C for 10 min, followed by 40 cycles of denaturation at 95 °C for 5 s, and annealing at 60 °C for 34 s each. The accumulation of PCR products was observed at each cycle by monitoring the increase in fluorescence of the reporter dye from dsDNA binding SYBR Green. After the PCR, the specificity of the amplification was assayed with the use of melting curves which were constructed in the range of 60 °C to 95 °C [29].

### 2.3. Analysis of Genetic Polymorphisms

#### 2.3.1. Sampling

For the analysis of genetic polymorphisms, five milliliters of venous blood was collected from each participant and transferred into a vacutainer containing 3% ethylenediaminetetraacetic acid (EDTA) to prevent coagulation. Samples were subsequently stored at −20 °C until DNA extraction.

#### 2.3.2. DNA Extraction from Blood Samples

Genomic DNA was extracted from blood samples using a commercially available DNA isolation kit (High Pure PCR Template Preparation Kit, Roche, Basel, Switzerland) according to the manufacturer’s instructions. The DNA concentration was assessed using a NanoDrop spectrophotometer (Thermo Fisher Scientific, Waltham, MA, USA).

#### 2.3.3. Real-Time PCR for SNPs Detection

Primer and probe sequences for *IL-1A*, *IL-1B* and *TNFA* polymorphisms (*IL-1A* −889: rs1800587; *IL-1B* +3954: rs1143634; *TNFA* −308: rs1800629) were obtained from Applied Biosystems, Waltham, MA, USA (assay ID for rs1800587: C_9546481_30; assay ID for rs1143634: C_9546517_10; assay ID for rs1800629: C_7514879_10).

Polymorphism detection was done using the ABI Prism 7500 Real-Time PCR System (Applied Biosystems, Waltham, MA, USA). Real-time PCR was carried out using a 25 µL reaction mix solution containing 12.50 µL 2xTaqMan^®^ Genotyping Master Mix (Thermo Fisher Scientific, Waltham, MA, USA), 1.25 µL SNP Assay, 2 µL of DNA. PCR cycling conditions included an initial denaturation at 95 °C for 10 min, followed by 40 cycles of 95 °C for 15 s and 60 °C for 1 min.

### 2.4. Statistical Analysis

Statistical analyses were performed using standard descriptive and inferential methods. The distribution of numerical variables was assessed for normality using the Kolmogorov–Smirnov test. As the data were not normally distributed, numerical variables are presented as median and interquartile range (IQR), whereas categorical variables are expressed as absolute numbers and percentages.

The prevalence of bacterial species and the genotype distribution in *IL-1A*, *IL-1B* and *TNFA* genes were compared using the Chi-square test or Fisher’s exact test, as appropriate, depending on expected cell frequencies.

The logistic regression models were conducted to examine whether genetic variants in selected genes predicted the presence of specific periodontopathogenic bacteria. *IL-1A*, *IL-1B*, and *TNFA* were entered as primary independent variables, and the presence of each bacterial species (*A. actinomycetemcomitans*, *P. gingivalis*, *P. intermedia*, *T. forsythia*, *T. denticola*; coded 1 for present, 0 for absent) was used as the outcome in five separate models. All models were adjusted for age (continuous), sex (female vs. male), systemic disease (yes/no), and smoking status (non-smoker vs. smoker) as potential confounders. Additional clinical variables, including snoring, number of natural teeth, number of implants, and periodontitis location, were initially examined in univariate logistic regression models, but none reached statistical significance and were therefore not included in the multivariable models given the sample size limitations. No further variable selection methods were applied, as all covariates were chosen based on clinical relevance.

Associations were expressed as odds ratios (ORs) with 95% confidence intervals (95% CI) and *p*-values based on Wald tests; model fit was evaluated with Nagelkerke’s R^2^, model χ^2^ *p*-value, and the area under the receiver operating characteristic curve (AUC).

All statistical tests were two-sided, with *p*-values < 0.05 considered statistically significant. Analyses were performed using R software (version 4.5.2, R Foundation for Statistical Computing, Vienna, Austria).

Hardy–Weinberg equilibrium (HWE) with the chi-square test was assessed for each SNP using an online Hardy–Weinberg Equilibrium Calculator [30]. A *p*-value > 0.05 was interpreted as no significant deviation from expected genotype distribution.

This study was conducted and reported in accordance with the STROBE guidelines for cross-sectional studies.

## 3. Results

### 3.1. Demographic and Clinical Characteristics of Patients

The analysis included 63 participants, whose demographic and clinical characteristics are summarized in Table 1. Slightly more participants were female (58.7%) than male (41.3%), although this difference was not statistically significant (*p* = 0.077). The median age was 57.0 years (IQR 43.5–67.0). Most individuals were free of systemic diseases (*n* = 42, 66.7%), while 21 (33.3%) had at least one condition. Among those with systemic conditions, elevated blood pressure was most prevalent (*n* = 17, 27.0%), followed by hypercholesterolemia (*n* = 6, 9.5%), and diabetes mellitus or rheumatoid arthritis (*n* = 2, 3.2% each). A few participants presented with multiple conditions, most commonly elevated blood pressure combined with one of the other disorders. Smoking was reported by 22 participants (34.9%), and 24 (38.1%) reported snoring. Participants retained a median of 21 natural teeth (IQR 9.5–26.0; range 0–32) and had a median of 4 implants (IQR 0.0–6.0; range 0–12), reflecting a wide range of oral status within the cohort. Regarding location of periodontitis, patients had a generalized (*n* = 55, 87.3%) or localized form (*n* = 8, 12.5%).

### 3.2. Distribution of Periodontopathogenic Bacteria Among Patients

Real-time PCR analysis detected all five target periodontopathogens in subgingival plaque samples, with bacterial load stratified into four categories (0: undetected; <10^4^ copies/plaque sample; 10^4^–10^6^ copies; >10^6^ copies).

**Figure 1:**
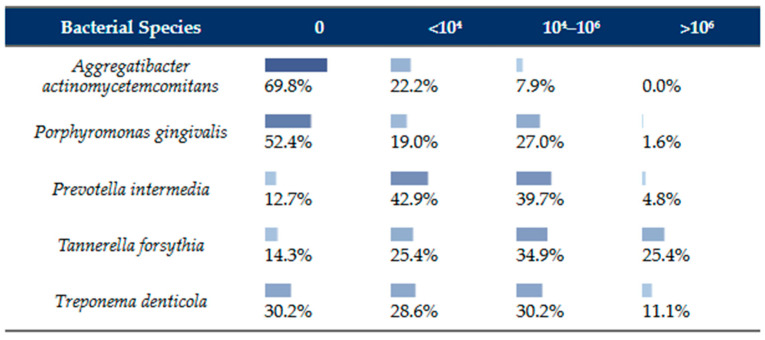
Detection levels of five periodontopathogenic bacteria in subgingival plaque samples from 63 patients with periodontal disease, as determined by real-time PCR. Bacterial loads are categorized as 0 (undetected), <10^4^ copies/plaque sample, 10^4^–10^6^ copies/plaque sample, and >10^6^ copies/plaque sample.

*A. actinomycetemcomitans* exhibited the lowest detection rate at 30.2% (19/63), predominantly at low loads (<10^4^ copies in 14 samples; 10^4^–10^6^ in 5), with no samples exceeding 10^6^ copies. *P. gingivalis* was detected in 47.6% (30/63 samples), mainly at moderate loads (10^4^–10^6^ in 17 samples, 10^4^ in 11 samples), and one sample at >10^6^ copies. *P. intermedia* showed high prevalence (87.3%, 55/63), distributed across low (27 samples), moderate (25 samples), and high loads (3 samples). *T. forsythia* had an 85.7% detection rate (54/63) and the highest bacterial burdens, with 16 samples >10^6^ copies, alongside 22 at 10^4^–10^6^ and 16 at <10^4^. *T. denticola* was detected in 69.8% of samples (44/63), with the majority at moderate loads (19 samples at 10^4^–10^6^ copies), followed by low loads (18 at <10^4^), and fewer at high loads (7 at >10^6^). These results indicate that *P. intermedia*, *T. forsythia*, and *T. denticola* were dominant periodontopathogens in this cohort, as seen in Figure 1.

### 3.3. Genotype and Allele Frequencies of Study Participants

Presence of *IL-1A*, *IL-1B* and *TNFA* polymorphisms and genotype distribution in study participants are presented in Table 2. The genotype frequencies of the three studied SNPs showed no significant deviation and confirmed to be in Hardy–Weinberg equilibrium (*p* > 0.05).

### 3.4. Distribution of IL-1A, IL-1B and TNFA Genotypes

The prevalence of bacterial species and genetic polymorphisms in *IL-1A*, *IL-1B*, and *TNFA* genes is summarized in Table 3. Overall, genetic polymorphisms varied between patients, with some differences statistically significant and others not. For *A. actinomycetemcomitans* and *P. intermedia*, genetic polymorphisms were similar between groups, with no significant differences. In *IL-1A* and *IL-1B* genes, CC genotype was significantly associated with increased loads of *P. gingivalis* (*p* = 0.032), whereas differences in genetic polymorphisms in *TNFA* were not significant. A higher prevalence of *T. forsythia* was significantly associated with CC genotype in *IL-1B* gene, while *IL-1A* and *TNFA* were not significantly different. For *T. denticola*, genetic polymorphisms in selected genes did not differ significantly between groups.

Five multivariable logistic regression models were constructed to evaluate whether genetic polymorphisms in *IL-1A*, *IL-1B*, and *TNFA* genes predict the presence of selected periodontal bacteria (*A. actinomycetemcomitans*, *P. gingivalis*, *P. intermedia*, *T. forsythia* and *T. denticola*) in patients from the Split–Dalmatia County, after adjustment for age, gender, smoking status, and systemic disease. Spearman correlation analysis demonstrated a strong positive association between *IL-1A* and *IL-1B* polymorphisms (ρ = 0.629, *p* < 0.001). To prevent multicollinearity and unstable coefficient estimates, genetic polymorphisms in *IL-1A* and *IL-1B* genes were not entered simultaneously into multivariable logistic regression models. Instead, separate models were fitted including either *IL-1A* or *IL-1B* genetic variant, together with the remaining covariates. Overall, the associations between genetic polymorphisms in selected genes and bacterial presence were species-specific, while demographic and clinical covariates showed limited predictive value Table 4.

No statistically significant associations were observed between *A. actinomycetemcomitans* or *P. intermedia* presence and any of the investigated genetic polymorphisms or covariates, indicating that the examined polymorphisms do not independently predict *A. actinomycetemcomitans* nor *P. intermedia* colonization in our sample. Increasing age was independently associated with a higher likelihood of *P. gingivalis* detection (OR = 1.06; *p* = 0.030). Importantly, carriers of the *IL-1A* CC genotype had a significantly increased probability of *P. gingivalis* presence compared with CT and TT genotype carriers (OR = 5.54; *p* = 0.009). No significant associations were found for *TNFA* polymorphism, smoking status, gender, or systemic disease. This model showed good overall performance, supporting a role of *IL-1A* genetic variability in susceptibility to *P. gingivalis* colonization. A strong and independent association was observed between *IL-1B* CC genotype and the presence of *T. forsythia*, with carriers exhibiting markedly higher odds of bacterial detection compared with CT and TT genotype carriers (OR = 8.48, *p* = 0.026). Other polymorphisms and covariates were not significant predictors. Among all models, this model showed the best fit and discriminatory capacity, indicating a potentially important role of *IL-1B* genetic variants in host susceptibility to *T. forsythia* (Nagelkerke R^2^ = 0.416; AUC = 0.886; model *p* = 0.011). Carriers of the *IL-1A* CC genotype had significantly higher odds of *T.denticola* presence compared with CT and TT genotype carriers (OR = 3.77; *p* = 0.041). No significant associations were identified for genetic polymorphisms in *IL-1B* and *TNFA* genes, or demographic and clinical variables. Although the overall model was not statistically significant, the observed association suggests a possible contribution of *IL-1A* polymorphism to colonization by this periodontal pathogen.

## 4. Discussion

In this population from Split–Dalmatia County, *IL-1A* and *IL-1B* genetic polymorphisms, but not *TNFA* polymorphism, were significantly associated with the presence of specific periodontopathogenic bacteria. Specifically, the *IL-1A* (−889 C/T) CC genotype significantly predicted the presence of *P. gingivalis* (*p* = 0.009) and *T. denticola* (*p* = 0.041), while the *IL-1B* (+3954 C/T) CC genotype significantly predicted the presence of *T. forsythia* (*p* = 0.026). These results suggest that host genetic variability in pro-inflammatory interleukin genes may contribute to bacterial colonization patterns in periodontal disease, highlighting the importance of host–microbe interactions in the pathogenesis of periodontitis.

Key pro-inflammatory cytokines, including IL-1α, IL-1β and TNF-α, play a central role in modulating the host inflammatory response to periodontopathogenic bacteria, contributing to tissue remodeling, and directly influencing alveolar bone resorption. Polymorphisms in the *IL-1A*, *IL-1B*, and *TNFA* genes have previously been suggested as genetic risk factors; however, results of previous studies have been inconsistent, likely due to differences in disease phenotype, ethnic structure, and methodology [20,26,31,32].

A recent meta-analysis reported that *IL-1A* −889 C/T T allele had a favorable association in the prevention of periodontitis risk with OR (95% CI), 1.12 (0.99–1.25). In contrast, IL −889 C/T C allele had a strong association with the periodontitis development with OR (95% CI), 0.75 (0.66–0.85) which is consistent with our results [33]. The results of the study conducted by Loo et al. demonstrate that the *IL-1A* CC genotype (OR = 4.368; 95% CI = 2.309 to 8.264) was significantly associated with periodontitis [34]. In our study, there was a significant association between *IL-1A* CC genotype carriers and increased probability of *P. gingivalis* and *T. denticola* presence. In contrast, meta-analysis conducted by Feng and Liu indicated that *IL-1A* −889 TT genotype was closely related to the susceptibility of chronic periodontitis in different populations, including the European population [18].

In their research Mesa et al. analyzed the association of polymorphisms in the *IL-1* gene cluster and the load of periodontopathogenic bacteria, where *IL-1A* C/T, T allele carriers presented significantly increased *P. intermedia* load [19]. Similarly, Stojanovska et al. demonstrated the association between *P. intermedia* and composite IL-1 genotype, but the significance was borderline (χ^2^ = 8.17; *p* = 0.06, *p* < 0.05). For the other tested periodontopathogens, there was no association between SNPs and bacteria loads [31]. Conversely, a case–control association study conducted on 415 northern European Caucasian patients with aggressive periodontitis and 874 healthy controls examined 10 SNPs in the genes of the IL1 cluster. The results showed no association between SNPs in the IL1 gene cluster and aggressive periodontitis [26]. Furthermore, Scapoli et al. also concluded that there is no association between generalized aggressive periodontitis and genetic variants in the IL1 gene cluster in an Italian Caucasian population [22]. Also, the Polish study did not find an association between periodontitis and *IL-1A*^−889^ and *IL-1B*^+3953^ TT genotype [35]. In our study, no statistically significant association was observed between four detected bacteria and genetic polymorphisms in *IL-1B* gene, which is shown in Table 4.

Considering high polymorphism within the *IL-1* gene, there is large individual variability in cytokine production. In particular, the SNP at position +3954 in the *IL-1B* gene was studied, where the T allele could be associated with a higher risk of developing inflammatory diseases. However, the results of previous studies are contradictory, as some studies associate the C allele with the development of the disease, while others were unable to determine any association.

According to the Dahash and Kusrat study in the Iraqi population, there was a significant decrease in IL-B +3954 TT genotype in periodontitis cases compared to the control group (*p* = 0.018), indicating that this genotype is associated with the reduced risk for periodontitis, and CC genotype with an increased risk for the disease development [36]. Likewise, Sharma et al. found a significant association between *IL-1B* +3954 CC genotype and chronic periodontitis [37]. Results of our study indicate that the CC genotype in *IL-1B* gene could potentially be a genetic risk factor for higher loads of *T. forsythia* and *P. gingivalis*, as shown in Table 3.

The results of Pani et al. study indicate an independent association of *IL-1B* +3954 T allele carriers and red complex bacterial species with increased IL-1β levels in gingival crevicular fluid of periodontitis sites, but their study included a small number of participants from different ethnic groups [20]. Also, some individual case–control studies associated *IL-1B* +3954 C/T polymorphism with periodontitis. In a Brazilian study, Ribeiro et al. concluded that the *IL-1B* +3954 T allele represented risk for chronic periodontitis (OR = 2.84; 95% CI = 1.44–5.62), particularly in smokers (OR = 4.43; 95% CI = 1.29–15.24; *p* = 0.025) and females (OR = 6.00; 95% CI = 2.13–16.93; *p* = 0.027) [38]. Additionally, Ayazi et al., in their pilot study, found the significant association between distribution of *IL-1B* genotypes and the risk of periodontal disease (*p* = 0.017) [39]. Their data suggest that *IL-1B* +3954 C/T polymorphisms could be potential candidates for genetic biomarkers of periodontitis. Although these studies associated *IL-1B* +3954 TT genotype with a higher likelihood of periodontitis development, meta-analysis do not support these findings, as there was no association between *IL-1B* +3954 polymorphism and periodontitis susceptibility, regardless of ethnicity [40]. Furthermore, Linhartova et al. could not find any association between *IL-1B* polymorphisms and aggressive periodontitis among adult subjects from Czech Republic [14], and no association between *IL-1B* +3954 TT genotype was seen in Greek patients [41]. These results were in line with our findings, as there was no association between *IL-1B* +3954 polymorphisms and the presence of four detected bacteria, shown in Table 4.

Our findings also show no association between *TNFA* (−308 G/A) polymorphisms and tested periodontopathogenic bacteria. The lack of association between *TNFA* polymorphisms and periodontitis was reported by Yücel et al. [42] and Costa et al. [43]. Additionally, the results of a recent meta-analysis showed no significant associations of the *TNFA* (−308 G/A) polymorphism with aggressive periodontitis in Caucasians [44]. However, the results of a Serbian study indicate that homozygous carriers of the A allele are significantly more prone to periodontitis [45]. Conversely, Ianni et al. reported an association between *TNFA* (−308 G/A) GG genotype and increased risk of periodontitis [46].

While genetic variants in the *TNFA* gene in certain populations were associated with increased susceptibility to periodontitis, the allele and genotype frequencies vary among populations, making it difficult to generalize results and require population-specific research.

The findings of the present study should be interpreted considering both its strengths and limitations. A major strength of this study is that it is, to our knowledge, the first investigation conducted in a Croatian population to evaluate the association between *IL-1A*, *IL-1B*, and *TNFA* genetic polymorphisms and the presence of key periodontopathogenic bacteria, with sensitive detection by real-time PCR and adjustment for age, gender, smoking, and systemic disease.

Nevertheless, several limitations should be acknowledged. The relatively small sample size limits statistical power, while the cross-sectional design precludes causal inference. In addition, recruitment from a single center and the lack of periodontally healthy controls may restrict the generalizability of the findings to the broader population. Although bacterial copy numbers were quantified, the main analyses were based on the presence/absence of bacteria to ensure model stability, which may obscure biologically meaningful gradients of colonization. Furthermore, detailed quantitative periodontal parameters, such as clinical attachment level, bleeding on probing, plaque index, and pocket depth distribution, were not included in the multivariable models and may act as potential confounders. Finally, only selected cytokine polymorphisms were analyzed, and other genetic or environmental factors may also contribute to periodontal bacterial colonization. These limitations highlight the need for larger, well-powered studies incorporating healthy controls, quantitative bacterial outcomes, and comprehensive clinical parameters.

## 5. Conclusions

In this cohort from Split-Dalmatia County, *IL-1A* CC genotype was associated with increased likelihood of *P. gingivalis* and *T. denticola* colonization, whereas the *IL-1B* CC genotype was independently associated with *T. forsythia* detection. These findings suggest that host genetic variability in pro-inflammatory interleukin genes may influence subgingival bacterial colonization patterns. Conversely, the T allele may confer a protective effect, being associated with lower loads of periodontopathogenic species. However, these findings should be interpreted with caution given the study limitations. Larger, longitudinal studies including periodontally healthy controls, a priori power calculations, quantitative bacterial load modeling, comprehensive clinical periodontal indices, and appropriate multiple testing adjustments are warranted to confirm and extend these observations.

## Figures and Tables

**Table 1:** Demographic and clinical characteristics of patients with periodontitis (*n* = 63).

Variable			*p* *
Gender; *n* (%)	male	26 (41.3)	0.077
	female	37 (58.7)	
Age (years); median (IQR)		57.0 (43.5–67.0)	/
Systemic diseases; *n* (%)	yes	21 (33.3)	0.006
	no	42 (66.7)	
Elevated blood pressure; *n* (%)	yes	17 (27.0)	<0.001
	no	46 (73.0)	
Smoking; *n* (%)	yes	22 (34.9)	0.011
	no	41 (65.1)	
Snoring; *n* (%)	yes	24 (38.1)	0.034
	no	39(61.9)	
Number of natural teeth; median (IQR)		21 (9.5–26.0)	/
Number of implants; median (IQR)		4 (0.0–6.0)	/
Location of periodontitis; *n* (%)	generalized	55 (87.3)	<0.001
	localized	8 (12.7)	

IQR—interquartile range; *—*p*-values for categorical variables were calculated using a one-sample chi-square test or Fisher’s exact test when expected counts were low; /—statistical test not applicable.

**Table 2:** Presence of *IL-1A*, *IL-1B* and *TNFA* polymorphisms and genotype distribution in the study participants.

Gene	SNP	Genotype	*n* (%)	Minor Allele	MAF (%)	Hardy–Weinberg Equilibrium	
						*p* Allele Frequency	χ^2^ *p* *
*IL-1A*	rs1800587	CC CT TT	31 (49.2) 28 (44.4) 4 (6.4)	T	28.57	0.71	0.50 0.48
*IL-1B*	rs1800587	CC CT TT	42 (66.6) 17 (27.0) 4 (6.4)	T	19.84	0.80	1.45 0.23
*TNFA*	rs1800629	GG GA AA	47 (74.6) 16 (25.4) 0 (0)	A	12.70	0.87	1.33 0.25

MAF—minor allele frequency; * *p*-values were calculated using the chi-square test.

**Table 3:** Prevalence of selected subgingival bacterial species and genetic polymorphisms in *IL-1A*, *IL-1B*, and *TNFA* genes in study participants (*n* = 63).

*IL-1A*		M	W (CC)	*p* *	Polymorphism genotype *n* (%)	
					heterozygous (CT)	homozygous (TT)
*A.* *actinomycetemcomitans*	+	8 (25%)	11 (35%)	0.365	5 (63%)	3 (38%)
	−	24 (75%)	20 (65%)		23 (96%)	1 (4%)
*P. gingivalis*	+	11 (34%)	19 (61%)	**0.032**	9 (82%)	2 (18%)
	−	21 (66%)	12 (39%)		19 (90%)	2 (10%)
*P.* *intermedia*	+	28 (88%)	27 (87%)	0.962	25 (89%)	3 (11%)
	−	4 (12%)	4 (13%)		3 (75%)	1 (25%)
*T.* *forsythia*	+	26 (81%)	28 (90%)	0.304	22 (85%)	4 (15%)
	−	6 (19%)	3 (10%)		6 (100%)	0 (0%)
*T.* *denticola*	+	19 (59%)	25 (81%)	0.066	16 (84%)	3 (16%)
	−	13 (41%)	6 (19%)		12 (92%)	1 (8%)
*IL-1B*		M	W (CC)	*p* *	Polymorphism genotype *n* (%)	
					heterozygous (CT)	homozygous (TT)
*A.* *actinomycetemcomitans*	+	6 (29%)	13 (31%)	0.846	3 (50%)	3 (50%)
	−	15 (71%)	29 (69%)		14 (93%)	1 (7%)
*P. gingivalis*	+	6 (29%)	24 (57%)	**0.032**	5 (83%)	1 (17%)
	−	15 (71%)	18 (43%)		12 (80%)	3 (20%)
*P.* *intermedia*	+	17 (81%)	38 (90%)	0.285	13 (76%)	4 (24%)
	−	4 (19%)	4 (10%)		4 (100%)	0 (0%)
*T.* *forsythia*	+	15 (71%)	39 (93%)	**0.022**	11 (73%)	4 (27%)
	−	6 (29%)	3 (7%)		6 (100%)	0 (0%)
*T.* *denticola*	+	13 (62%)	31 (73%)	0.332	11 (85%)	2 (15%)
	−	8 (38%)	11 (27%)		6 (75%)	2 (25%)
*TNFA*		M	W (GG)	*p* *	Polymorphism genotype *n* (%)	
					heterozygous (GA)	homozygous (AA)
*A.* *actinomycetemcomitans*	+	4 (25%)	15 (32%)	0.603	4 (100%)	0 (0%)
	−	12 (75%)	32 (68%)		12 (100%)	0 (0%)
*P. gingivalis*	+	5 (31%)	25 (53%)	0.129	5 (100%)	0 (0%)
	−	11 (69%)	22 (47%)		11 (100%)	0 (0%)
*P.* *intermedia*	+	15 (94%)	40 (85%)	0.370	15 (100%)	0 (0%)
	−	1 (6%)	7 (15%)		1 (100%)	0 (0%)
*T.* *forsythia*	+	16 (100%)	38 (81%)	0.059	16 (100%)	0 (0%)
	−	0 (0%)	9 (19%)		0 (100%)	0 (0%)
*T.* *denticola*	+	13 (81%)	31 (65%)	0.250	13 (100%)	0 (0%)
	−	3 (19%)	16 (35%)		3 (100%)	0 (0%)

M—mutated; W—wild-type; +/− (positive/negative presence of selected periodontal bacteria: *A. actinomycetemcomitans*, *P. gingivalis*, *P. intermedia*, *T. forsythia* and *T. denticola*); * *p*-values for categorical variables were calculated using the chi-square test.

**Table 4:** Multivariable logistic regression models assessing the association between genetic polymorphisms in *IL-1A*, *IL-1B* and *TNFA* genes and the presence of periodontopathogenic bacteria, adjusted for demographic, lifestyle, and systemic factors (*n* = 63).

Variables in the Model	*A. actinomycetemcomitans* OR (95% CI); *p*-Value	*P. gingivalis* OR (95% CI); *p*-Value	*P. intermedia* OR (95% CI); *p*-Value	*T. forsythia* OR (95% CI); *p*-Value	*T. denticola* OR (95% CI); *p*-Value
Age (years)	1.03 (0.98–1.09); 0.256	1.06 (1.01–1.12); **0.030**	1.00 (0.94–1.07); 0.990	0.96 (0.89–1.04); 0.359	1.03 (0.98–1.08); 0.246
Female (ref. male)	2.64 (0.76–9.18); 0.126	0.90 (0.28–2.83); 0.850	0.80 (0.17–3.87); 0.782	0.71 (0.09–5.61); 0.741	0.73 (0.22–2.41); 0.604
Systemic disease (ref. none)	0.26 (0.05–1.29); 0.099	0.62 (0.14–2.74); 0.527	0.81 (0.11–5.88); 0.833	0.28 (0.03–2.31); 0.238	0.82 (0.17–3.95); 0.805
Non-smokers (ref. smokers)	0.91 (0.26–3.21); 0.878	1.62 (0.47–5.52); 0.444	0.63 (0.11–3.70); 0.607	1.05 (0.10–10.68); 0.965	0.89 (0.25–3.12); 0.852
*IL-1A* CC genotype (ref. CT/TT)	2.18 (0.64–7.48); 0.213	5.54 (1.54–19.97); **0.009**	0.96 (0.19–4.85); 0.962	—	3.77 (1.06–13.45); **0.041**
*IL-1B* CC genotype (ref. CT/TT)	—	—	—	8.48 (1.30–55.47); **0.026**	—
*TNFA* GG genotype (ref. GA/AA)	1.38 (0.34–5.60); 0.649	2.33 (0.59–9.09); 0.226	0.37 (0.04–3.38); 0.377	~0 (0–∞); 0.994	0.31 (0.07–1.47); 0.141
Model performance					
Nagelkerke R2	0.151	0.270	0.042	0.416	0.155
*p*-value	0.311	0.027	0.964	0.011	0.294
AUC	0.706	0.757	0.678	0.886	0.722

Each column represents a separate multivariable logistic regression model with the presence of the indicated periodontopathogenic bacterium as the dependent variable. *IL-1A* and *IL-1B* genetic variants were not included simultaneously in the same model due to collinearity.

## Data Availability

The data presented in this study are available upon request from the corresponding author.
